# Factors associated with HIV viral load “blips” and the relationship between self-reported adherence and efavirenz blood levels on blip occurrence: a case–control study

**DOI:** 10.1186/s12981-016-0100-4

**Published:** 2016-03-22

**Authors:** Aaron Farmer, Xun Wang, Anuradha Ganesan, Robert G. Deiss, Brian K. Agan, Thomas A. O’Bryan, Kevin Akers, Jason F. Okulicz

**Affiliations:** Infectious Disease Service, San Antonio Military Medical Center, 3551 Roger Brooke Drive, Fort Sam Houston, TX USA; Infectious Disease Clinical Research Program, Department of Preventive Medicine and Biostatistics, Uniformed Services University of the Health Sciences, Bethesda, MD USA; Walter Reed National Military Medical Center, Bethesda, MD USA; Division of Infectious Diseases, Naval Medical Center of San Diego, San Diego, CA USA; United States Army Institute of Surgical Research, Fort Sam Houston, TX USA; Henry M. Jackson Foundation for the Advancement of Military Medicine, Inc., Bethesda, MD USA

**Keywords:** Self-reported adherence, Human immunodeficiency virus, Blips

## Abstract

**Background:**

The uncertain etiology of HIV viral load (VL) blips may lead to increased use of clinical resources. We evaluated the association of self-reported adherence (SRA) and antiretroviral (ART) drug levels on blip occurrence in US Military HIV Natural History Study (NHS) participants who initiated the single-tablet regimen efavirenz/emtricitabine/tenofovir disoproxil fumarate (EFV/FTC/TDF).

**Methods:**

ART-naïve NHS participants started on EFV/FTC/TDF between 2006 and 2013 who achieved VL suppression (<50 copies/mL) within 12 months and had available SRA and stored plasma samples were included. Participants with viral blips were compared with those who maintained VL suppression without blips. Untimed EFV plasma levels were evaluated on consecutive blip and non-blip dates by high performance liquid chromatography, with a level ≥1 mcg/mL considered therapeutic. SRA was categorized as ≥85 or <85 %. Descriptive statistics were performed for baseline characteristics and univariate and multivariate Cox proportional hazard models were used to assess the relationship between covariates and blip occurrence.

**Results:**

A total of 772 individuals met inclusion criteria, including 99 (13 %) blip and 673 (87 %) control participants. African-American was the predominant ethnicity and the mean age was 29 years for both groups. SRA ≥ 85 % was associated with therapeutic EFV levels at both blip and non-blip time points (P = 0.0026); however no association was observed between blips and SRA or EFV levels among cases. On univariate analysis of cases versus controls, blips were associated with higher mean pre-treatment VL (HR 1.45, 95 % CI 1.11–1.89) and pre-treatment CD4 count <350 cells/µL (68.1 vs 49.7 %). Multivariate analysis also showed that blips were associated with a higher mean VL (HR 1.42, 95 % CI 1.08–1.88; P = 0.0123) and lower CD4 count at ART initiation, with CD4 ≥500 cells/µL having a protective effect (HR 0.45, 95 % CI 0.22–0.95; P = 0.0365). No association was observed for demographic characteristics or SRA.

**Conclusion:**

Blips are commonly encountered in the clinical management of HIV-infected patients. Although blip occurrence was not associated with SRA or EFV blood levels in our study, blips were associated with HIV-related factors of pre-ART high VL and low CD4 count. Additional studies are needed to determine the etiology of blips in HIV-infected patients.

## Background

The principal goal of antiretroviral therapy (ART) is to achieve long-term maintenance of VL suppression during the course of HIV treatment [1]. VL “blips” are defined as transient, low-level increases in HIV viral load followed by return to suppression without a change in therapy [2–4]. The appearance of blips may result in greater use of resources, such as more frequent VL monitoring, and can lead to concerns about the efficacy and durability of the ART regimen [2, 5]. The underlying etiology for blips is uncertain. Proposed explanations for the appearance of blips include transient increases in viral production due to fluctuations in adherence [6], concurrent illnesses or vaccinations [7, 8], and artifact due to variability in the VL assay [9–11]. Another possibility, with considerable clinical implications, is that blips may represent ongoing low-level viral replication and may ultimately result in virologic failure and the development of drug resistance [10, 12–15].

A previous study of 194 ART-experienced patients with blips on multi-tablet regimens (MTRs), anchored by protease inhibitors (PIs) or non-nucleoside reverse transcriptase inhibitors (NNRTIs), found a marginal association between blips and self-reported adherence (SRA) [16]. However, no association between blip frequency and blood levels of PIs or NNRTIs was observed and assessments of NRTI concentrations were not performed. In contrast, a separate study using MTRs noted decreased adherence the week prior to blip appearance [6]. One limiting factor to both of these studies was the use of MTRs rather than single-tablet regimens (STRs) since there may be differential adherence to the individual medications in MTR regimens [17–19]. These limitations in MTR studies make it challenging to determine a potential association between adherence and drug levels in the appearance of blips.

The STR era commenced in 2006 with the US Food and Drug Administration (FDA) approval of the first single-tablet regimen efavirenz/emtricitabine/tenofovir disoproxil fumarate (EFV/FTC/TDF) [20]. STRs have major advantages over MTRs for the study of blips. First, reported adherence is inclusive of all medications in the regimen. Second, obtaining therapeutic drug levels for a single drug infers adherence to all components of the regimen, thereby eliminating the potential for differential adherence to regimen components and reducing cost of drug level assessments.

To minimize or eliminate the potential confounders of MTR regimens in prior blip studies, we retrospectively analyzed the occurrence of blips in US Military HIV Natural History Study (NHS) participants who initiated the single-tablet regimen EFV/FTC/TDF. The first aim of this study was to evaluate the factors associated with blip occurrence in NHS participants on EFV/FTC/TDF. The second aim was to evaluate correlation of SRA and untimed EFV blood levels from repository specimens for those experiencing blips.

## Methods

The NHS is a prospective observational study of active duty US military members and beneficiaries infected with HIV. Participants are evaluated approximately every 6–12 months with data collected for demographics, medical diagnoses, laboratory monitoring, and HIV treatment outcomes. All participants enrolled provided written informed consent for this IRB-approved study and were ≥18 years of age.

The NHS database was queried for ART-naïve participants treated with the single-tablet regimen EFV/FTC/TDF between 2006 and 2013 and achieved VL suppression to <50 copies/mL within 12 months of ART initiation. Participants were divided into two groups, based on the presence or absence of blips during follow-up, in order to analyze the association of demographic and HIV-related factors with blip observations. Both the blip group and control group (no blips) maintained virologic suppression for at least 1 year. Blips were defined as transient VL values ≥50 copies/mL, but less than 1000 copies/mL, with return to <50 copies/mL without a change in therapy.

Inclusion criteria for those in the blip group included both SRA assessment and repository plasma available on the date of blip occurrence as well as an additional timepoint either immediately before or after the blip date. Only the first blip episode was evaluated per patient. Adherence was evaluated using a standardized questionnaire at each clinical visit. The total percentage of adherence was assigned by the patient-reported percentage of doses missed within the last month. This questionnaire further classified any missed doses by time periods from greater than 1 month up to prior to the day of the visit. For data analysis, self-reported adherence was categorized as < or ≥85 %, as this lower limit has been associated with maintenance of viral suppression [17]. Each subgroup was then analyzed at blip and non-blip time periods. SRA for controls was measured at the first available time point ≥1 year after ART initiation.

Untimed efavirenz levels were measured by high-performance liquid chromatography (HPLC) by the US Army Institute of Surgical Research laboratory at Fort Sam Houston, TX. Efavirenz was purchased from Sigma Aldrich (St. Louis, MO). Methanol and acetonitrile were HPLC grade and purchased from VWR (Radnor, PA). Ultrapure deionized water was generated using an Aqua Solutions Type I water purification system (resistivity ≥ 18.2 MΩ) and used in all applications. Efavirenz stocks were prepared in methanol and then added to blank human plasma (Biological Specialty Corporation, Colmar, PA) as previously described [21]. Standards were prepared at 0.05, 0.1, 0.5, 1, 5, and 10 µg/mL and assayed in 250 µL aliquots. A full calibration curve was constructed at the beginning and end of the sample set, and their average values were used to assign concentrations to the subject samples. Two sets of validation standards at 0.05, 0.5, and 10 µg/mL were prepared in mobile phase and used to assess repeatability of the assay.

Efavirenz was quantified using a Dionex UltiMate 3000 HPLC (Thermo Fisher) according to the protocol described by Veldcamp et al. [21]. For efavirenz HPLC, the limit of detection (LOD) for the assay was 0.05 µg/mL. The limit of quantification (LOQ), set at 10 times the LOD, was therefore 0.5 µg/mL. The average percent recovery for the five standard concentrations measured was 92 %. The variation of the assay was analyzed using validation standards in mobile phase. The coefficient of determination (R^2^) values for the two calibration curves were 0.94 and 0.99. A level ≥1 mcg/mL was considered therapeutic per DHHS guidelines [1] and EFV levels were dichotomized to therapeutic or subtherapeutic if above or below this threshold, respectively. These levels were obtained from the same specimens used for viral load to avoid discrepancies between different samples.

Descriptive statistics were performed to evaluate the baseline characteristics of the study population. All categorical variables were evaluated with either Fisher’s exact test or the Chi square test when appropriate, and all continuous variables were tested by using Wilcoxon–Mann–Whitney test. Univariate and multivariate Cox proportional hazard models evaluated the association of blips and other covariates. All univariate factors were included in the multivariate models and removed in a stepwise fashion based on an alpha of 0.05. The final models were a priori adjusted for age at ART initiation, race (categorized as Caucasian, African-American, and Other), viral load and CD4 count at ART initiation, and SRA (categorized as < or ≥85 %). Hazard ratios (HR) are reported with 95 % confidence intervals (CI). All tests were two-sided, and a P value less than 0.05 was considered statistically significant. Statistical analyses were performed using SAS version 9.4 (SAS Institute, Cary, NC).

## Results

A total of 772 participants met inclusion criteria, including 99 in the blip group and 673 in the control group (Table 1). The majority of participants were male and the mean age at HIV diagnosis was similar in both groups (approximately 29 years). African-American was the most common race for both groups (49.5 and 41.3 %, respectively). The mean viral load at diagnosis was 4.6 log_10_copies/mL and 4.4 log_10_copies/mL (P = 0.0497) for blip and non-blip groups, respectively. Compared to controls at ART start, the blip group had a lower mean CD4 count (284 ± 190 vs. 353 ± 189 cells/µL; P = 0.0002) and higher mean log_10_ VL (4.8 ± 0.9 vs. 4.5 ± 1.0 copies/mL; P = 0.0017). The mean viral load at blip was 76 copies/mL (SD ± 52). The mean time from seroconversion to first ART was not significantly different (1.7 ± 2.8 vs 1.7 ± 2.5 years; P = 0.8564).

**Table 1:** Baseline characteristics of NHS participants

Characteristic	All	Blip group	Control group	P value
Number, n	772	99	673	
Mean age at HIV diagnosis (years)	29.1 (12.6)	29.8 (12.4)	29.1 (12.8)	0.4881
Mean time from HIV negative to seroconversion (years)	0.6 (0.6)	0.7 (0.6)	0.6 (0.6)	0.1704
Mean time from seroconversion to first ART (years)	1.7 (2.5)	1.7 (2.8)	1.7 (2.5)	0.8564
Male	724 (93.8)	94 (94.9)	630 (93.6)	
Female	48 (6.2)	5 (5.1)	43 (6.4)	
Race				0.1599
Caucasian	305 (39.5)	38 (38.4)	267 (39.7)	
African-American	327 (42.4)	49 (49.5)	278 (41.3)	
Other	140 (18.1)	12 (12.1)	128 (19.0)	
Mean CD4 count at HIV diagnosis (cells/µL)	437 (273)	432 (308)	439 (265)	0.2258
CD4 count at HIV diagnosis (cells/µL)				0.4664
<350	214 (30.7)	33 (36.3)	181 (29.9)	
350 to <500	213 (30.6)	25 (27.5)	188 (31.1)	
≥500	269 (38.6)	33 (36.3)	236 (39.0)	
Mean CD4 count at ART (cells/µL)	340 (194)	284 (190)	353 (189)	0.0002
CD4 count at ART (cells/µL)				0.0022
<350	369 (52.1)	64 (68.1)	305 (49.7)	
350 to <500	212 (29.9)	22 (23.4)	190 (30.9)	
≥500	127 (17.9)	8 (8.5)	119 (19.4)	
Mean viral load at ART (log_10_copies/mL)	4.5 (1.0)	4.8 (0.9)	4.5 (1.0)	0.0017

Data expressed as N (%) or mean (SD)

*ART* antiretroviral therapy

SRA was ≥85 % for 84 of 99 blip patients (84.8 %) and 513 of 673 controls (76.2 %) (Table 2). When the time period was dichotomized into a missed dose last week or greater than 1 week ago, the majority of participants in each group reported a missed dose as occurring greater than 1 week ago [71 patients for blips (82.6 %) and 450 patients for controls (84.1 %)]. Only 19 patients (21.8 %) in the blip and 114 patients (21.3 %) in the control group reported missing any doses in the past 2 weeks.

**Table 2:** Self-reported adherence

Characteristic	All	Blip group	Control group	P value
Total self-reported adherence (%)				0.1603
≥85	597 (96.2)	84 (97.7)	513 (96.1)	
<85	23 (3.8)	2 (2.3)	21 (3.9)	
Last time missed a dose				0.2097
Last week	100 (16.1)	15 (17.4)	85 (15.9)	
Longer than last week	521 (83.9)	71 (82.6)	450 (84.1)	
Missed a dose in the last weekend				0.1528
No	572 (93.2)	80 (93.0)	492 (93.2)	
Yes	42 (6.8)	6 (7.0)	36 (6.8)	
Total missed doses in the last 2 weeks				0.1297
0	489 (78.6)	68 (78.2)	421 (78.7)	
1 or more	127 (20.4)	17 (19.5)	110 (20.6)	
All doses	5 (0.8)	2 (2.3)	3 (0.6)	
Don’t know	1 (0.2)	0 (0.0)	1 (0.2)	

Data expressed as N (%) or mean (SD)

Of the 198 blip and non-blip specimens analyzed (no efavirenz samples obtained from the control group), 16 (8 %) had EFV levels below therapeutic levels, including 7 of 99 (7 %) blip and 9 of 99 (9 %) non-blip time points (P = 0.77). Among those with subtherapeutic EFV levels, adherence ≥85 % was self-reported by 6 of 7 (85 %) and 8 of 9 (88 %) participants on blip and non-blip dates, respectively (P = 1.00). SRA ≥85 % was associated with therapeutic EFV levels at both blip and non-blip time points (P = 0.0026).

In univariate analyses for blip participants compared to controls (Table 3), higher mean VL at ART initiation (HR 1.45, 95 % CI 1.11–1.89) and greater percentage of pre-treatment CD4 count <350 cells/µL (68.1 vs 49.7 %) were associated with blips. On multivariate analysis including demographic and HIV characteristics as well as adherence (Table 4), higher mean VL (HR 1.42, 95 % CI 1.08–1.88) at ART initiation was significantly associated with blip occurrence. A CD4 count greater ≥500 showed a protective effect against blip occurrence (HR 0.45, 95 % CI 0.22–0.95).

**Table 3:** Unadjusted cox proportional hazard model for blip participants compared to controls

	Blip group N (%) or mean (SD)	Control group N (%) or mean (SD)	Univariate hazard ratio (95 % CI)	P value
Number, n	99	673		
Demographics				
Mean age at ART (years)	34.4 (14.9)	32.9 (13.4)	1.00 (0.98,1.02)	0.9756
Mean age at HIV diagnosis (years)	29.8 (12.4)	29.1 (12.8)	1.00 (0.98,1.02)	0.7467
Gender				
Male	94 (94.9)	630 (93.6)	Ref	
Female	5 (5.1)	43 (6.4)	0.66 (0.27,1.63)	0.3735
Race				
Caucasian	38 (38.4)	267 (39.7)	Ref	
African-American	49 (49.5)	278 (41.3)	1.22 (0.80,1.86)	0.3632
Other	12 (12.1)	128 (19.0)	0.73 (0.38,1.39)	0.3332
Time from HIV negative to seroconversion (years)	0.7 (0.6)	0.6 (0.6)	1.24 (1.04,1.49)	0.0186
Time from seroconversion to first ART (years)	1.7 (2.8)	1.7 (2.5)	1.02 (0.95,1.11)	0.536
Viral load at HIV diagnosis (log_10_copies/mL)	4.6 (0.9)	4.4 (1.0)	1.32 (0.99,1.76)	0.0577
Viral load at ART initiation (log_10_copies/mL)	4.8 (0.9)	4.5 (1.0)	1.45 (1.11,1.89)	0.0064
Viral load at ART Initiation	69,368 (115,313)	32,281 (86,112)	1.00 (1.00,1.00)	0.3596
CD4 cell count at HIV diagnosis (cells/mL)	432 (308)	439 (265)	1.00 (1.00,1.00)	0.2717
CD4 cell count at HIV diagnosis (cells/mL)				
<350	33 (36.3)	181 (29.9)	Ref	
350 to <500	25 (27.5)	188 (31.1)	0.74 (0.44,1.24)	0.2563
≥500	33 (36.3)	236 (39.0)	0.78 (0.48,1.27)	0.3223
CD4 cell count at ART initiation (cells/mL)	284 (190)	353 (189)	1.00 (1.00,1.00)	0.0553
CD4 cell count at ART initiation (cells/mL)				
<350	64 (68.1)	305 (49.7)	Ref	
350 to <500	22 (23.4)	190 (30.9)	0.61 (0.38,0.99)	0.0472
≥500	8 (8.5)	119 (19.4)	0.39 (0.19, 0.82)	0.0131
HIV medication adherence history				
Self-reported antiretroviral drug adherence (%)				
≥85	96 (98.0)	637 (96.2)	Ref	
<85	2 (2.0)	25 (3.8)	0.52 (0.13, 2.10)	0.358
Self-reported last time missed a dose				
Last week	16 (16.3)	98 (14.8)	Ref	
Longer than last week	82 (83.7)	563 (85.2)	0.88 (0.52,1.51)	0.6532
Self-reported missed a dose in the last weekend				
Yes	3 (3.1)	45 (6.8)	Ref	
No	95 (96.9)	612 (93.2)	0.44 (0.14,1.40)	0.166
Self-reported total missed doses in the last 2 weeks				
0	80 (81.6)	529 (80.0)	Ref	
1 or more	18 (18.4)	126 (19.1)	0.94 (0.56,1.57)	0.809
All	0 (0.0)	4 (0.6)		
Don’t know	0 (0.0)	2 (0.3)		

Data expressed as N (%) or mean (SD)

*ART* antiretroviral therapy

**Table 4:** Adjusted cox proportional hazard model for blip participants compared to controls

Characteristics	Blip group N (%) or mean (SD)	Control group N (%) or mean (SD)	Multivariate hazard ratio (95 % CI)	P value
Number, n	99	673		
Mean age at ART (years)	34.4 (14.9)	32.9 (13.4)	1 (0.98,1.02)	0.9737
Race				
Caucasian	38 (38.4)	267 (39.7)	Ref	
African-American	49 (49.5)	278 (41.3)	1.14 (0.73,1.78)	0.5773
Other	12 (12.1)	128 (19.0)	0.68 (0.35,1.35)	0.2727
Viral load at ART initiation (log_10_ copies/mL)	4.8 (0.9)	4.5 (1.0)	1.42 (1.08,1.88)	0.0123
CD4 cell count at ART initiation (cells/µL)				
0–350	64 (68.1)	305 (49.7)	Ref	
350 to <500	22 (23.4)	190 (30.9)	0.64 (0.39,1.05)	0.0793
≥500	8 (8.5)	119 (19.4)	0.45 (0.22, 0.95)	0.0365
Self-reported antiretroviral drug adherence (%)				
≥85	96 (98.0)	637 (96.2)	Ref	
<85	2 (2.0)	25 (3.8)	0.5 (0.12, 2.04)	0.3335

Data expressed as N (%) or mean (SD)

*ART* antiretroviral therapy

## Discussion

In this study, we sought to evaluate adherence and the occurrence of VL blips in patients taking a once-daily STR. The etiology of blips is unclear with prior studies reporting conflicting data on the effect of adherence [5, 6, 22]. In this highly adherent population, there was no significant association between blip occurrence and SRA or EFV levels. These results suggest that blip observations can be explained by factors other than self-reported adherence and blood levels of ART.

Initial studies evaluating blips and adherence used multi-drug, multi-tablet regimens, which may have affected the ability to draw accurate conclusions [2, 5, 6]. With the increasing availability of STRs, it has been shown that adherence is increased compared to MTRs, even in difficult to treat populations [17, 19]. In addition, a meta-analysis of once versus twice daily therapy showed a difference in favor of the once daily regimen, where the mean adherence for STRs in treatment-naïve patients was 82–99.8 % [18]. Based on these studies, we used the single-tablet regimen EFV/FTC/TDF to avoid potential confounding of differential adherence for different tablets in the regimen, and our observed adherence of 97 % by SRA and >95 % by drug levels is consistent with prior results [15].

Although SRA has been shown to have high sensitivity (95 %) and positive predictive value (84 %) for adherence in comparison with untimed drug levels, we chose to measure both in this study, as overestimation by SRA has been previously reported [23, 24]. No significant differences were shown between adherence by SRA or blood levels. Although the levels for efavirenz were untimed, this is unlikely to have affected the results given the long half-life of the drug (40–50 h), thus negating the need for timing relative to administration of the dose. Of note, in one study evaluating the utility of untimed drug levels for efavirenz, 13 (37 %) of 35 subjects had levels <1 mcg/mL but did not show evidence of virologic failure suggesting that a broader therapeutic range may be necessary [25].

Given the validity of SRA and drug levels, combined with the high adherence in this population, this study provides additional support that blips are unlikely to be associated with lack of adherence. Alternative hypotheses suggest that blips may be secondary to assay variability, decreased assay sensitivity at lower viral load or release from a latent reservoir [10, 11, 26]. In a study by Murray et al. [10], HIV RNA and total and episomal HIV DNA were assessed using a single copy assay with a lower limit of quantification (LLOQ) of <1 copy/mL and the Amplicor assay with a LLOQ of 50 copies/mL. Based on their comparison of the single copy assay to the Amplicor assay, they surmised that blips were due to under-reporting of the Amplicor assay and therefore represent true elevations in plasma viral load. Total HIV DNA in peripheral blood was also measured and did not show any increase suggestive of activation of latently infected cells. Since the specific VL assays used in our cohort varied over time and by clinical site, the impact of various assays on blip occurrence could not be assessed in our study.

Vaccinations and intercurrent infection have also been studied as possible causes of blips but with conflicting results [7, 27]. While these studies suggested these factors may have an impact on the occurrence of blips, a review published in 2006 noted that most studies were small and retrospective, potentially limiting the significance of the findings [28]. Further evidence against vaccination and intercurrent illness as the etiology of blips was found in a study consisting of frequent viral load sampling every 2–3 days which did not reveal any correlation between the occurrence of blips and either of these elements [5].

Several studies have also shown no association between host and viral factors such as CD4 cell count, duration of infection/suppression or pre-treatment VL [3, 5]. This is in contrast to our results, where we found a statistically significant association in multivariate analysis between a lower CD4 count and higher viral load at baseline and the development of blips. DiMascio et al. [29] similarly found viral blip frequency to be inversely correlated with higher baseline CD4 count and not with reduced adherence. Differences in patient populations may help explain the conflicting results between studies. For instance, the characteristics of the patients in the DiMascio study were similar to those in our cohort. However, the patients studied by Sklar and Hofstra had higher baseline CD4 counts but similar median viral loads [3, 30].

Furthermore, our NHS participants are unique in several aspects. The high adherence observed in this study is not unexpected as the cohort was composed of US military personnel and beneficiaries with historically high (>90 %) rates of SRA [31]. These participants are provided with free access to healthcare including prescriptions and regular visits to HIV clinicians which may minimize many of the factors shown to impact adherence in studies in other populations. For example, STR adherence can vary in many groups including populations supported by managed care organizations (73 % achieving ≥90 % adherence) and HIV-infected women (adherence increased from 78 to 85 % on STR) [32, 33]. Typical characteristics that negatively affect adherence in other studies are not typically present or are mitigated in our population including intravenous drug use and homelessness. These factors are less common in the NHS compared to the general population and even compared to the Veterans Affairs system which has a similar government-sponsored healthcare system [34]. In addition, simply being evaluated by a health care provider has been shown to have a positive impact on adherence and NHS study participants are typically seen at least every 6 months [35]. Other significant strengths of our study include the use of an STR and the occurrence of blips even with the high level of adherence observed, further supporting the hypothesis that blips are unlikely to be attributed to suboptimal adherence.

Our study has several limitations. First, the high overall adherence may have limited the ability to detect any difference between the blip and control group. Also the use of untimed efavirenz levels did not allow for formal assessment of pharmacokinetics. As noted above however, the long half-life of the medication and overall high adherence in this population likely minimize any impact from these “random” drug levels. Ultrasensitive genotype testing could not be performed to evaluate for drug resistance mutations due to the limited volume of repository samples. However, resistance is unlikely to explain blips as these episodes were followed by return to VL suppression without changes in therapy. Finally, different viral load assays were used during the study period and the potential contribution of various VL assay types could not be ascertained.

## Conclusion

In conclusion, we observed no association between SRA or drug levels in the occurrence of VL blips and adherence. Other studies have shown that persistent low level viremia may also be a factor, with blips being an artifact of assay limitation. Further study into clinical outcomes and significance of detectable viremia, particularly at a level less than the DHHS-defined cutoff for virologic failure of <200 copies/mL, are needed to define the impact of these findings. In current clinical practice, blips will continue to be observed, even in populations with high adherence, and additional studies investigating alternate etiologies are needed to fully understand the occurrence of blips.

## Abbreviations

ART: antiretroviral therapy
VL: viral load
SRA: self-reported adherence
NHS: Natural History Study
EFV/FTC/TDF: efavirenz, emtricitabine, tenofovir
MTR: multi-tablet regimen
STR: single-tablet regimen
PI: protease inhibitors
NNRTI: non-nucleoside reverse transcriptase inhibitors
HPLC: high-performance liquid chromatography
LOD: limit of detection
LOQ: limit of quantification
